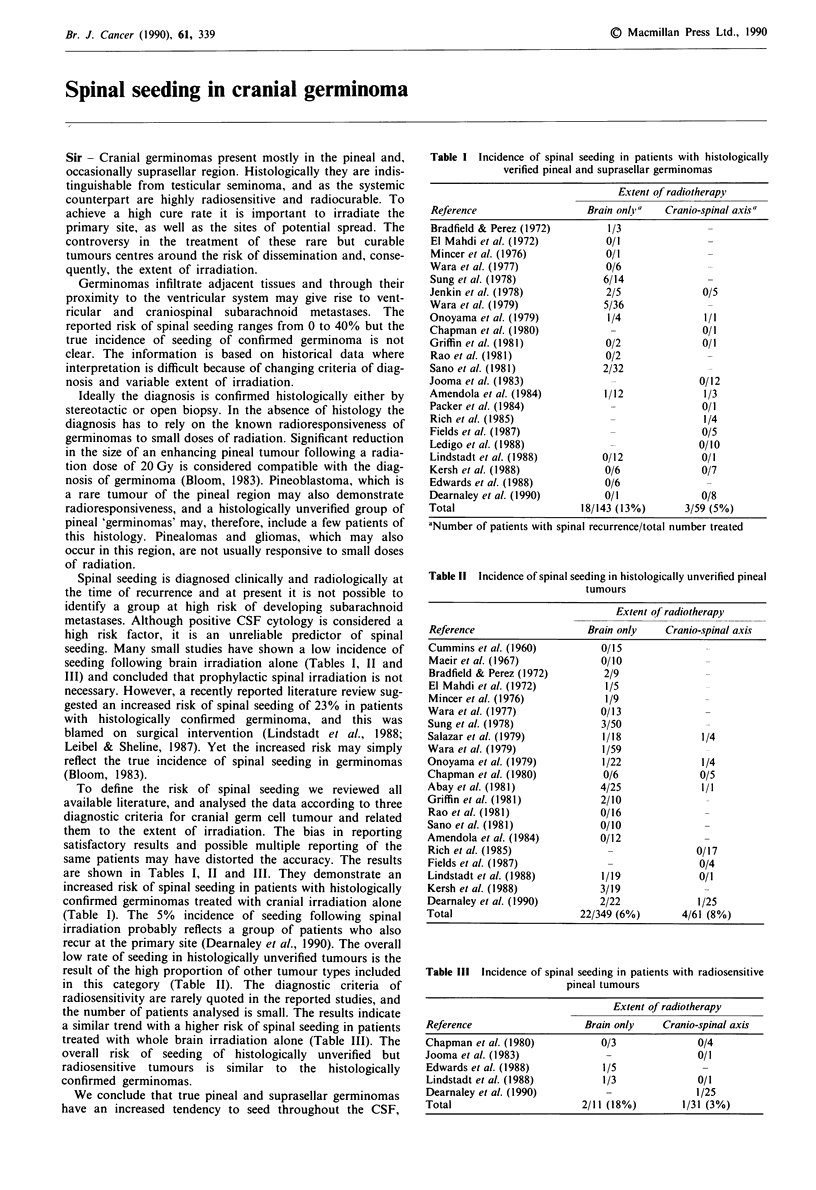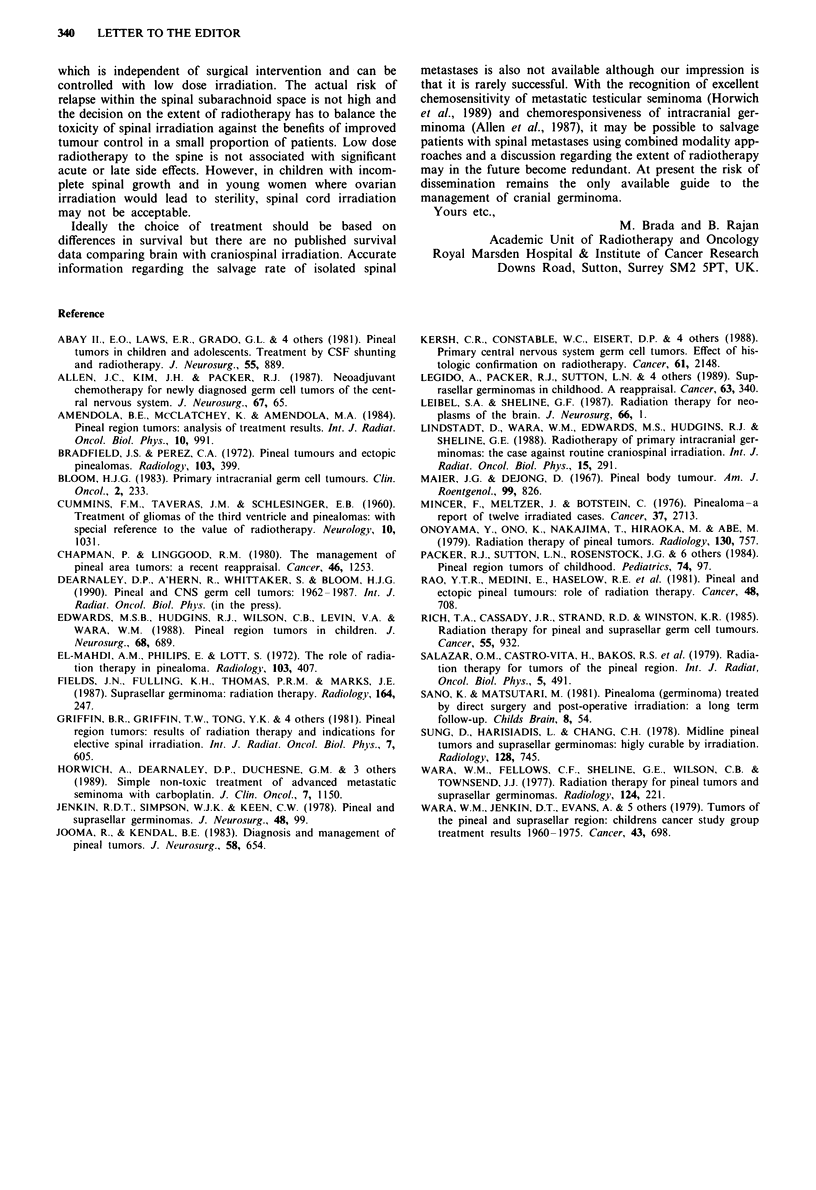# Spinal seeding in cranial germinoma.

**DOI:** 10.1038/bjc.1990.69

**Published:** 1990-02

**Authors:** M. Brada, B. Rajan


Br. J. Cancer (1990), 61, 339 © Macmillan Press Ltd., 1990

### Spinal seeding in cranial germinoma

**Sir** – Cranial germinomas present mostly in the pineal and, occasionally suprasellar region. Histologically they are indistinguishable from testicular seminoma, and as the systemic counterpart are highly radiosensitive and radiocurable. To achieve a high cure rate it is important to irradiate the primary site, as well as the sites of potential spread. The controversy in the treatment of these rare but curable tumours centres around the risk of dissemination and, consequently, the extent of irradiation.

Germinomas infiltrate adjacent tissues and through their proximity to the ventricular system may give rise to ventricular and craniospinal subarachnoid metastases. The reported risk of spinal seeding ranges from 0 to 40% but the true incidence of seeding of confirmed germinoma is not clear. The information is based on historical data where interpretation is difficult because of changing criteria of diagnosis and variable extent of irradiation.

Ideally the diagnosis is confirmed histologically either by stereotactic or open biopsy. In the absence of histology the diagnosis has to rely on the known radioresponsiveness of germinomas to small doses of radiation. Significant reduction in the size of an enhancing pineal tumour following a radiation dose of 20 Gy is considered compatible with the diagnosis of germinoma (Bloom, 1983). Pineoblastoma, which is a rare tumour of the pineal region may also demonstrate radioresponsiveness, and a histologically unverified group of pineal 'germinomas' may, therefore, include a few patients of this histology. Pinealomas and gliomas, which may also occur in this region, are not usually responsive to small doses of radiation.

Spinal seeding is diagnosed clinically and radiologically at the time of recurrence and at present it is not possible to identify a group at high risk of developing subarachnoid metastases. Although positive CSF cytology is considered a high risk factor, it is an unreliable predictor of spinal seeding. Many small studies have shown a low incidence of seeding following brain irradiation alone (Tables I, II and III) and concluded that prophylactic spinal irradiation is not necessary. However, a recently reported literature review suggested an increased risk of spinal seeding of 23% in patients with histologically confirmed germinoma, and this was blamed on surgical intervention (Lindstadt *et al.*, 1988; Leibel & Sheline, 1987). Yet the increased risk may simply reflect the true incidence of spinal seeding in germinomas (Bloom, 1983).

To define the risk of spinal seeding we reviewed all available literature, and analysed the data according to three diagnostic criteria for cranial germ cell tumour and related them to the extent of irradiation. The bias in reporting satisfactory results and possible multiple reporting of the same patients may have distorted the accuracy. The results are shown in Tables I, II and III. They demonstrate an increased risk of spinal seeding in patients with histologically confirmed germinomas treated with cranial irradiation alone (Table I). The 5% incidence of seeding following spinal irradiation probably reflects a group of patients who also recur at the primary site (Dearnaley *et al.*, 1990). The overall low rate of seeding in histologically unverified tumours is the result of the high proportion of other tumour types included in this category (Table II). The diagnostic criteria of radiosensitivity are rarely quoted in the reported studies, and the number of patients analysed is small. The results indicate a similar trend with a higher risk of spinal seeding in patients treated with whole brain irradiation alone (Table III). The overall risk of seeding of histologically unverified but radiosensitive tumours is similar to the histologically confirmed germinomas.

We conclude that true pineal and suprasellar germinomas have an increased tendency to seed throughout the CSF,

**Table I** Incidence of spinal seeding in patients with histologically verified pineal and suprasellar germinomas

| Reference | Extent of radiotherapy: Brain only[a] | Cranio-spinal axis[a] |
|---|---|---|
| Bradfield & Perez (1972) | 1/3 | – |
| El Mahdi *et al.* (1972) | 0/1 | – |
| Mincer *et al.* (1976) | 0/1 | – |
| Wara *et al.* (1977) | 0/6 | – |
| Sung *et al.* (1978) | 6/14 | – |
| Jenkin *et al.* (1978) | 2/5 | 0/5 |
| Wara *et al.* (1979) | 5/36 | – |
| Onoyama *et al.* (1979) | 1/4 | 1/1 |
| Chapman *et al.* (1980) | – | 0/1 |
| Griffin *et al.* (1981) | 0/2 | 0/1 |
| Rao *et al.* (1981) | 0/2 | – |
| Sano *et al.* (1981) | 2/32 | – |
| Jooma *et al.* (1983) | – | 0/12 |
| Amendola *et al.* (1984) | 1/12 | 1/3 |
| Packer *et al.* (1984) | – | 0/1 |
| Rich *et al.* (1985) | – | 1/4 |
| Fields *et al.* (1987) | – | 0/5 |
| Ledigo *et al.* (1988) | – | 0/10 |
| Lindstadt *et al.* (1988) | 0/12 | 0/1 |
| Kersh *et al.* (1988) | 0/6 | 0/7 |
| Edwards *et al.* (1988) | 0/6 | – |
| Dearnaley *et al.* (1990) | 0/1 | 0/8 |
| Total | 18/143 (13%) | 3/59 (5%) |

[a]Number of patients with spinal recurrence/total number treated

**Table II** Incidence of spinal seeding in histologically unverified pineal tumours

| Reference | Extent of radiotherapy: Brain only | Cranio-spinal axis |
|---|---|---|
| Cummins *et al.* (1960) | 0/15 | – |
| Maeir *et al.* (1967) | 0/10 | – |
| Bradfield & Perez (1972) | 2/9 | – |
| El Mahdi *et al.* (1972) | 1/5 | – |
| Mincer *et al.* (1976) | 1/9 | – |
| Wara *et al.* (1977) | 0/13 | – |
| Sung *et al.* (1978) | 3/50 | – |
| Salazar *et al.* (1979) | 1/18 | 1/4 |
| Wara *et al.* (1979) | 1/59 | – |
| Onoyama *et al.* (1979) | 1/22 | 1/4 |
| Chapman *et al.* (1980) | 0/6 | 0/5 |
| Abay *et al.* (1981) | 4/25 | 1/1 |
| Griffin *et al.* (1981) | 2/10 | – |
| Rao *et al.* (1981) | 0/16 | – |
| Sano *et al.* (1981) | 0/10 | – |
| Amendola *et al.* (1984) | 0/12 | – |
| Rich *et al.* (1985) | – | 0/17 |
| Fields *et al.* (1987) | – | 0/4 |
| Lindstadt *et al.* (1988) | 1/19 | 0/1 |
| Kersh *et al.* (1988) | 3/19 | – |
| Dearnaley *et al.* (1990) | 2/22 | 1/25 |
| Total | 22/349 (6%) | 4/61 (8%) |

**Table III** Incidence of spinal seeding in patients with radiosensitive pineal tumours

| Reference | Extent of radiotherapy: Brain only | Cranio-spinal axis |
|---|---|---|
| Chapman *et al.* (1980) | 0/3 | 0/4 |
| Jooma *et al.* (1983) | – | 0/1 |
| Edwards *et al.* (1988) | 1/5 | – |
| Lindstadt *et al.* (1988) | 1/3 | 0/1 |
| Dearnaley *et al.* (1990) | – | 1/25 |
| Total | 2/11 (18%) | 1/31 (3%) |

which is independent of surgical intervention and can be controlled with low dose irradiation. The actual risk of relapse within the spinal subarachnoid space is not high and the decision on the extent of radiotherapy has to balance the toxicity of spinal irradiation against the benefits of improved tumour control in a small proportion of patients. Low dose radiotherapy to the spine is not associated with significant acute or late side effects. However, in children with incomplete spinal growth and in young women where ovarian irradiation would lead to sterility, spinal cord irradiation may not be acceptable.

Ideally the choice of treatment should be based on differences in survival but there are no published survival data comparing brain with craniospinal irradiation. Accurate information regarding the salvage rate of isolated spinal metastases is also not available although our impression is that it is rarely successful. With the recognition of excellent chemosensitivity of metastatic testicular seminoma (Horwich *et al.*, 1989) and chemoresponsiveness of intracranial germinoma (Allen *et al.*, 1987), it may be possible to salvage patients with spinal metastases using combined modality approaches and a discussion regarding the extent of radiotherapy may in the future become redundant. At present the risk of dissemination remains the only available guide to the management of cranial germinoma.

Yours etc.,

M. Brada and B. Rajan
Academic Unit of Radiotherapy and Oncology
Royal Marsden Hospital & Institute of Cancer Research
Downs Road, Sutton, Surrey SM2 5PT, UK.

### Reference

ABAY II., E.O., LAWS, E.R., GRADO, G.L. & 4 others (1981). Pineal tumors in children and adolescents. Treatment by CSF shunting and radiotherapy. *J. Neurosurg.*, **55**, 889.

ALLEN, J.C., KIM, J.H. & PACKER, R.J. (1987). Neoadjuvant chemotherapy for newly diagnosed germ cell tumors of the central nervous system. *J. Neurosurg.*, **67**, 65.

AMENDOLA, B.E., MCCLATCHEY, K. & AMENDOLA, M.A. (1984). Pineal region tumors: analysis of treatment results. *Int. J. Radiat. Oncol. Biol. Phys.*, **10**, 991.

BRADFIELD, J.S. & PEREZ, C.A. (1972). Pineal tumours and ectopic pinealomas. *Radiology*, **103**, 399.

BLOOM, H.J.G. (1983). Primary intracranial germ cell tumours. *Clin. Oncol.*, **2**, 233.

CUMMINS, F.M., TAVERAS, J.M. & SCHLESINGER, E.B. (1960). Treatment of gliomas of the third ventricle and pinealomas: with special reference to the value of radiotherapy. *Neurology*, **10**, 1031.

CHAPMAN, P. & LINGGOOD, R.M. (1980). The management of pineal area tumors: a recent reappraisal. *Cancer*, **46**, 1253.

DEARNALEY, D.P., A'HERN, R., WHITTAKER, S. & BLOOM, H.J.G. (1990). Pineal and CNS germ cell tumors: 1962–1987. *Int. J. Radiat. Oncol. Biol. Phys.* (in the press).

EDWARDS, M.S.B., HUDGINS, R.J., WILSON, C.B., LEVIN, V.A. & WARA, W.M. (1988). Pineal region tumors in children. *J. Neurosurg.*, **68**, 689.

EL-MAHDI, A.M., PHILIPS, E. & LOTT, S. (1972). The role of radiation therapy in pinealoma. *Radiology*, **103**, 407.

FIELDS, J.N., FULLING, K.H., THOMAS, P.R.M. & MARKS, J.E. (1987). Suprasellar germinoma: radiation therapy. *Radiology*, **164**, 247.

GRIFFIN, B.R., GRIFFIN, T.W., TONG, Y.K. & 4 others (1981). Pineal region tumors: results of radiation therapy and indications for elective spinal irradiation. *Int. J. Radiat. Oncol. Biol. Phys.*, **7**, 605.

HORWICH, A., DEARNALEY, D.P., DUCHESNE, G.M. & 3 others (1989). Simple non-toxic treatment of advanced metastatic seminoma with carboplatin. *J. Clin. Oncol.*, **7**, 1150.

JENKIN, R.D.T., SIMPSON, W.J.K. & KEEN, C.W. (1978). Pineal and suprasellar germinomas. *J. Neurosurg.*, **48**, 99.

JOOMA, R., & KENDAL, B.E. (1983). Diagnosis and management of pineal tumors. *J. Neurosurg.*, **58**, 654.

KERSH, C.R., CONSTABLE, W.C., EISERT, D.P. & 4 others (1988). Primary central nervous system germ cell tumors. Effect of histologic confirmation on radiotherapy. *Cancer*, **61**, 2148.

LEGIDO, A., PACKER, R.J., SUTTON, L.N. & 4 others (1989). Suprasellar germinomas in childhood. A reappraisal. *Cancer*, **63**, 340.

LEIBEL, S.A. & SHELINE, G.F. (1987). Radiation therapy for neoplasms of the brain. *J. Neurosurg*, **66**, 1.

LINDSTADT, D., WARA, W.M., EDWARDS, M.S., HUDGINS, R.J. & SHELINE, G.E. (1988). Radiotherapy of primary intracranial germinomas: the case against routine craniospinal irradiation. *Int. J. Radiat. Oncol. Biol. Phys.*, **15**, 291.

MAIER, J.G. & DEJONG, D. (1967). Pineal body tumour. *Am. J. Roentgenol.*, **99**, 826.

MINCER, F., MELTZER, J. & BOTSTEIN, C. (1976). Pinealoma–a report of twelve irradiated cases. *Cancer*, **37**, 2713.

ONOYAMA, Y., ONO, K., NAKAJIMA, T., HIRAOKA, M. & ABE, M. (1979). Radiation therapy of pineal tumors. *Radiology*, **130**, 757.

PACKER, R.J., SUTTON, L.N., ROSENSTOCK, J.G. & 6 others (1984). Pineal region tumors of childhood. *Pediatrics*, **74**, 97.

RAO, Y.T.R., MEDINI, E., HASELOW, R.E. *et al.* (1981). Pineal and ectopic pineal tumours: role of radiation therapy. *Cancer*, **48**, 708.

RICH, T.A., CASSADY, J.R., STRAND, R.D. & WINSTON, K.R. (1985). Radiation therapy for pineal and suprasellar germ cell tumours. *Cancer*, **55**, 932.

SALAZAR, O.M., CASTRO-VITA, H., BAKOS, R.S. *et al.* (1979). Radiation therapy for tumors of the pineal region. *Int. J. Radiat, Oncol. Biol. Phys.*, **5**, 491.

SANO, K. & MATSUTARI, M. (1981). Pinealoma (germinoma) treated by direct surgery and post-operative irradiation: a long term follow-up. *Childs Brain*, **8**, 54.

SUNG, D., HARISIADIS, L. & CHANG, C.H. (1978). Midline pineal tumors and suprasellar germinomas: higly curable by irradiation. *Radiology*, **128**, 745.

WARA, W.M., FELLOWS, C.F., SHELINE, G.E., WILSON, C.B. & TOWNSEND, J.J. (1977). Radiation therapy for pineal tumors and suprasellar germinomas. *Radiology*, **124**, 221.

WARA, W.M., JENKIN, D.T., EVANS, A. & 5 others (1979). Tumors of the pineal and suprasellar region: childrens cancer study group treatment results 1960–1975. *Cancer*, **43**, 698.